# Intracerebral transplantation of erythropoietin‐producing fibroblasts facilitates neurogenesis and functional recovery in an ischemic stroke model

**DOI:** 10.1002/brb3.1274

**Published:** 2019-03-28

**Authors:** Pin‐Chun Chou, Yi‐Chieh Tsai, Shiu‐Jau Chen, Li‐Kai Tsai, Chung‐Liang Chien

**Affiliations:** ^1^ Graduate Institute of Anatomy and Cell Biology, College of Medicine National Taiwan University Taipei Taiwan; ^2^ Department of Neurology and Stroke Center National Taiwan University Hospital and National Taiwan University College of Medicine Taipei Taiwan; ^3^ Department of Medicine Mackay Medical College New Taipei City Taiwan

**Keywords:** cell therapy, erythropoietin, fibroblast, ischemic stroke, neurogenesis

## Abstract

**Introduction:**

Erythropoietin (EPO) can enhance neurogenesis and fibroblasts can secrete growth factors; together, they may benefit ischemic stroke. We transplanted EPO‐producing fibroblasts into the rodent infarcted brain to test their effect on neurogenesis and functional recovery.

**Methods:**

A total of 10^6^ cells of EPO‐producing NIH/3T3 fibroblasts (EPO/EGFP/3T3) or enhanced green fluorescence protein (EGFP)‐expressing fibroblasts (EGFP/3T3) were stereotaxically injected into the infarcted striatum of adult rats that received transient middle cerebral artery occlusion (MCAO) surgery 1 day poststroke. On day 14 after MCAO, the animals were euthanized for the evaluation of neurogenesis via immunohistochemistry and of the expression of growth factors using enzyme‐linked immunosorbent assay. The infarct volume was analyzed using magnetic resonance imaging and the neurological behavior was assessed using the neurological severity scoring performed within 14 days after MCAO.

**Results:**

The MCAO rats with EPO/EGFP/3T3 treatment showed high EPO expression in the infarcted brain for at least 1 week. The concentration of brain‐derived neurotrophic factor was higher in both hemispheres of MCAO rats with either EGFP/3T3 or EPO/EGFP/3T3 treatment at 14 days poststroke compared with untreated MCAO rats. The number of Ki‐67‐, nestin‐, or doublecortin‐immunoreactive cells in bilateral subventricular zones was higher in EPO/EGFP/3T3‐treated MCAO rats than it was in untreated MCAO control animals, indicating the enhancement of neurogenesis after EPO/EGFP/3T3 treatment. Notably, post‐MCAO EPO/EGFP/3T3 treatment significantly reduced infarct size and improved functional recovery.

**Conclusion:**

The intracerebral transplantation of EPO‐producing fibroblasts benefited an ischemic stroke model probably via the enhancement of neurogenesis.

## 1 INTRODUCTION

Stroke is one of the leading causes of mortality and physical/mental disability worldwide (Benjamin et al., 2017). Regarding ischemic stroke, the current standard treatments, which include thrombolytic therapy and endovascular thrombectomy, only benefit a small group of patients, and most patients who survive stroke suffer from long‐term functional deficits (Jung et al., 2010; Sugawara & Chan, 2003). Although poststroke neuroprotective therapy has been investigated for decades, unfortunately no treatment has shown obvious beneficial effects in clinical trials (Charidimou et al., 2017). The blood–brain barrier (BBB), which protects the brain from systemic toxicity, may prevent the penetration of drugs into brain tissues. Therefore, intracerebral delivery of certain treatments, particularly those with multiple therapeutic mechanisms, might provide an alternative direction for future stroke therapy.

Erythropoietin (EPO), a well‐known hematopoietic cytokine, has various pleiotropic effects, such as the promotion of neovascularization, the mobilization of endothelial progenitor cells, and the induction of antiapoptotic and anti‐inflammatory processes (Brines et al., 2000; Chong, Kang, & Maiese, 2003). Although preclinical studies have demonstrated that systemic EPO treatment facilitated stroke recovery in experimental stroke models (Gonzalez et al., 2013; Nguyen, Cherry, Scott, Ryou, & Mallet, 2014; Siren et al., 2001; Wang, Zhang, Wang, Zhang, & Chopp, 2004), clinical trials using systemic EPO administration did not consistently show effectiveness and safety in stroke patients (Yao et al., 2017). Systemic delivery of high‐dose EPO is required to overcome its poor BBB penetration (Alnaeeli et al., 2012; Zhang et al., 2014) and achieve sufficient brain targeting; however, this approach may increase the risk of systemic thromboembolism (Kirkeby et al., 2008; Meng et al., 2011; Siren et al., 2001).

Fibroblasts are relatively resistant to hypoxic environments (Shinde & Frangogiannis, 2014) and secret several neurotrophic factors, such as the brain‐derived neurotrophic factor (BDNF), the vascular endothelial growth factor (VEGF), and the nerve growth factor (NGF) (Dudas et al., 2011; Saito, Hamasaki, & Shibuya, 2003; Young et al., 1975). Therefore, in this study, we attempted to use a fibroblast cell line as a carrier and transplant EPO‐producing fibroblasts directly into the infarcted brain of a rodent model of ischemic stroke. The aim of this study was to investigate the therapeutic effect of the intracerebral transplantation of EPO‐producing fibroblasts on endogenous neurogenesis and poststroke functional recovery.

## 2 MATERIALS AND METHODS

### 2.1 Cell preparation

An EPO‐ and enhanced green fluorescence protein (EGFP)‐producing NIH/3T3 fibroblast cell line (EPO/EGFP/3T3) and an EGFP‐expressing NIH/3T3 cell line (EGFP/3T3) were generated as described previously (Li, Chen, & Chien, 2015). Cells were cultured in Dulbecco's modified Eagle's medium (DMEM, Gibco, Waltham, MA) supplemented with 10% fetal bovine serum (FBS, Gibco), 1% nonessential amino acids, and 1% antibiotic–antimycotic solution (Gibco) in a 37°C humidified incubator with 5% CO_2_. EPO/EGFP/3T3 or EGFP/3T3 cells were tripsinized and collected in phosphate‐buffered saline (PBS) just before cell transplantation.

### 2.2 MCAO and stereotaxic intracerebral transplantation

An ischemic stroke model with transient middle cerebral artery occlusion (MCAO) was used as described previously, with modifications (Tsai et al., 2011). Briefly, adult male Sprague Dawley rats (225–260 g) were anesthetized via intraperitoneal injection of Telazol (25 mg/kg) and Xylazine (10 mg/kg) and normal nonlabored breathing was maintained throughout the surgery. After exposure of the right carotid artery in a supine posture, an MCAO suture (MSRC37B280PK50, RWD Life Science, San Diego, CA) was inserted into the right common carotid artery, passed along the internal carotid artery, and brought to the orifice of the right middle cerebral artery (MCA) at a depth of 1.9 cm, to occlude the blood flow of the right MCA. After 1 hr of MCAO, the MCAO suture was withdrawn, to allow reperfusion of the right MCA. The animal was then placed back in its cage (day 0) and received stereotaxic intracerebral cell transplantation on the second day after MCAO (day 1).

**Figure 1:**
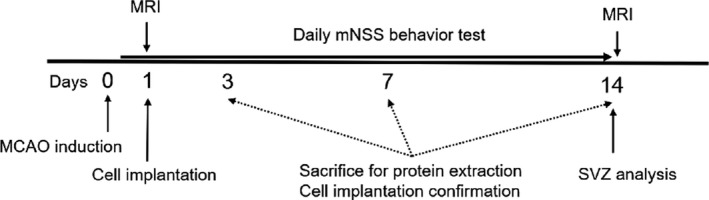
Schematic outline of the study

For cell transplantation, the MCAO rats were anesthetized again and fixed in the stereotactic apparatus (Kopf Instruments, Tujunga, CA). A hole was drilled through the skull, for cell injection. A total of 10^6^ cells in 8 μl of PBS were slowly injected into the infarcted brain at the right striatum (anteroposterior, 0.5 mm; mediolateral, 2.5 mm; dorsoventral, 5.0 mm from the bregma) using a Hamilton syringe (Hamilton Robotics, Reno, NV) for 5 min. The cells were transplanted into the right striatum near the subventricular zone (SVZ), to trigger the stem cell niche in this zone. The needle was then withdrawn and the incision was sutured. In the 2 weeks that followed the procedure, the animals underwent a magnetic resonance imaging (MRI) study and behavioral assessment and were then sacrificed for histological and molecular analyses (Figure 1). The procedures were approved by the Institutional Animal Care and Use Committee (IACUC‐20170124) of the National Taiwan University College of Medicine and the College of Public Health, Taiwan.

### 2.3 Protein extraction and ELISA

On days 3, 7, and 14 after MCAO, the animals underwent transcardial PBS perfusion and the brain was removed. Bilateral striata were dissected using an anatomic microscope (Olympus CH‐2; Olympus), homogenized in cold lysis buffer (8 M urea, 1% Triton X‐100, 300 mM Na_2_PO_4_, 0.1% 2‐mercaptoethanol, and protease inhibitors), incubated on ice for 15 min, sonicated, and centrifuged at 14,000 rpm at 4°C for 15 min. Supernatants were then collected and the protein concentration was measured based on the Bradford protein assay (Bio‐Rad, Hercules, CA).

To obtain quantitative data for growth factors secreted in the striatum after MCAO, the following enzyme‐linked immunosorbent assay (ELISA) kits were used: Erythropoietin Quantikine ELISA Kit (R&D System, Minneapolis, MN), BDNF E_max_^®^ ImmunoAssay System (Promega, Madison, WI), VEGF ELISA (RayBiotech, Norcross, GA), and beta‐NGF ELISA (RayBiotech). All procedures were performed according to the manufacturers’ manuals. After the reaction was terminated using STOP solution, the optical density was determined at 450 nm on a microplate reader (Ultrospec^® ^3100 Pro, Amersham Bioscience, Piscataway, NJ).

### 2.4 Immunohistochemistry

On days 7 and 14 after MCAO, the animals were sacrificed by transcardial perfusion with 4% paraformaldehyde (PFA) and the brain was removed, cut into 2‐mm‐thick slices, postfixed in 4% PFA in PBS overnight, and cryoprotected in 30% sucrose solution for 7 days. Brains were then embedded in optimal cutting temperature compound (Leica, Wetzlar, Germany) and rapidly frozen in isobutane with dry ice. Frozen brains were sliced into 14‐μm‐thick coronal sections on a cryostat (Leica CM3050). Brain sections were fixed with methanol, permeated with 1% Triton–PBS for 30 min, blocked with 3% FBS, and incubated with primary antibodies at 4°C overnight. The sections were then treated with secondary antibodies and Hoechst 33342 (1:1,000; Invitrogen, Waltham, MA) for 1 hr. Finally, sections were washed and mounted with Fluoro‐Gel (Electron Microscopy Sciences, Hatfield, PA), and images were acquired using a Leica TCS SP5 confocal microscope. The primary antibodies used in this experiment included a rabbit polyclonal anti‐Ki‐67 antibody (1:300; Abcam, Cambridge, MA), a mouse monoclonal anti‐nestin antibody (1:1,000, Abcam), a rabbit polyclonal anti‐doublecortin (DCX) antibody (1:500; Abcam), a mouse monoclonal anti‐glial fibrillary acidic protein (GFAP) antibody (1:200; Sigma‐Aldrich, St. Louis, MO), and a rabbit polyclonal anti‐green fluorescence protein (GFP) antibody (1:400; Abcam). The secondary antibodies used were Alexa 488 donkey anti‐rabbit and Alexa 594 donkey anti‐mouse antibodies (1:200; Molecular Probes, Waltham, MA).

Immunoreactivities for Ki‐67, nestin, DCX, and GFAP were determined in bilateral SVZs. Coronal forebrain sections between bregma 0.3–1.3 mm were analyzed. The number of cells was semiquantitively estimated based on an image analysis approach. Confocal microscopy was used to scan the vertical center of the stained section with a 1.8 μm thickness per step and five steps per section. The resulting projection image was converted to grayscale, a similar threshold was set for all images, and the area of specific immunoreactivity was measured using LAS X (Leica) and image J. Since Ki67 immunoreactive signals appear in nucleus colocalized with Hoechst 33342, we can count the number of Ki67 immunopositive cells directly. Ki‐67 immunoreactivity was thus calculated as the total number of Ki‐67 immunoreactive cells over the total number of cells in the SVZ area. For Nestin, DCX, and GFAP immunoreactivities, we measured the immunoreactive areas using the software of image J and the immunoreactivities were calculated as the immunopositive area over the SVZ area.

### 2.5 Magnetic resonance imaging

On day 1 (before cell transplantation) and day 14 after MCAO, rats were anesthetized with 2% isofluorane. The body core temperature was maintained at 37°C by a heated circulating water pad. Rats were then placed in a stereotaxic holder and mounted on a 72 mm volume (transmit)/25 mm surface (receive) radio frequency coil ensemble for MRI (Bruker, Billerica, MA). The MRI experiments were performed on a horizontal bore 7 Tesla scanner operating on a Bruker Avance platform.

Infarct volume was evaluated by T2‐weighted imaging on days 1 and 14 post‐MCAO. A total of 15 T2‐weighted axial slices (0.5 mm thick) encompassing the entire damaged area were collected, with a total imaging time of 10 min (field‐of‐view [FOV], 25.6 × 25.6 mm; matrix size, 256 × 256; in‐plane resolution, 256 μm; echo time [TE], 50 ms; repetition time [TR], 3,000 ms; echo train length, 16; number of averages [NA], 3). Diffusion‐weighted imaging (DWI) was also used to confirm the development of cerebral infarct on day 1 post‐MCAO (FOV, 25.6 × 25.6 mm; diffusion gradient duration, 4 ms; b value, 1,000 m/Tms; direction, 3; TE, 30 ms; TR, 4,500 ms; ∆, 15 ms; NA, 4). T2‐weighted images were processed and analyzed using the ImageJ system with the Bruker toolbox. The infarct volume is presented as the ratio of the infarct area over the whole brain area.

### 2.6 Behavioral tests

The animals were subjected to a modified Neurological Severity Score (mNSS) test (Supplementary Material) on the day before MCAO surgery (day 0) and daily after surgery for 14 days in all groups (Chen et al., 2001). Neurological function was graded on a scale of 0 to 18 (normal score, 0; maximal deficit score, 18). Different tests for motor (raising test, walking test, and posture), sensation (placing test and proprioceptive test), and reflex abnormalities (pinna, corneal, and startle reflex) were performed to evaluate the functional outcome of the animals.

### 2.7 Statistical analysis

All data were presented as mean values ± SEM and plotted using GraphPad Prism^®^ 7.0 (GraphPad, La Jolla, CA). We used one‐way ANOVA followed by Tukey's post hoc comparison test for statistical analysis using the GraphPad software (GraphPad Prism version 7.00). Significance was set at *p* < 0.05.

## 3 RESULTS

### 3.1 Implantation of EPO/EGFP/3T3 cells in the infarcted brain

**Figure 2:**
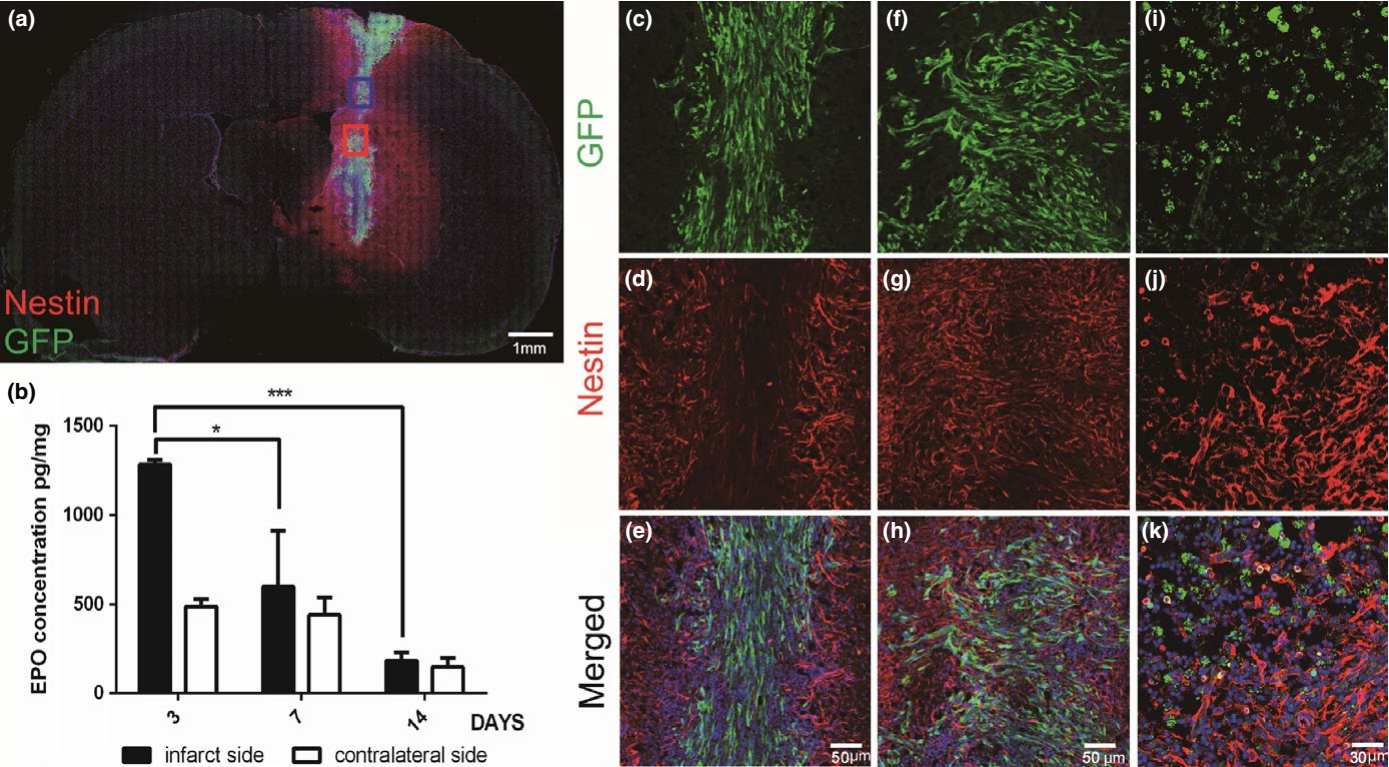
Appearance of transplanted EPO/EGFP/3T3 cells in middle cerebral artery occlusion (MCAO) rats. (a) In MCAO rats that received intracerebral EPO/EGFP/3T3 treatment, coronal brain sections collected on post‐MCAO day 7 were immunostained with anti‐GFP (green, implanted EPO/EGFP/3T3 cells) and anti‐nestin (red, neural stem/progenitor cells) antibodies and counterstained with Hoechst 33342 (blue). The GFP‐immunoreactive cells were distributed in the right striatum and cortex along the stereotaxic needle tract and were surrounded by nestin‐immunoreactive cells. Scale bar, 1 mm. (b) The concentration of erythropoietin (EPO) in bilateral striata was determined using enzyme‐linked immunosorbent assay (ELISA) on post‐MCAO days 3, 7, and 14. In the infarcted striatum, the concentration of EPO was higher on post‐MCAO day 3 than it was on post‐MCAO days 7 and 14 (*n* = 3 in each group; *p < 0.05; ***p < 0.001). (c–h) The GFP‐immunoreactive cells showed a typical spindle morphology on post‐MCAO day 7. Scale bar, 50 μm. (i–k) On post‐MCAO day 14, the number of GFP‐immunoreactive cells was reduced and cells became fragmented. Scale bar, 30 μm

To demonstrate that the EPO/EGFP/3T3 cells were successfully transplanted into the infarcted brain, we used an anti‐GFP antibody to identify the implanted cells in brain sections. On post‐MCAO day 7, the GFP‐immunoreactive cells were distributed in the right striatum and cortex along the stereotaxic needle tract (Figure 2a) and exhibited a typical spindle morphology (Figure 2c,f). In addition, nestin‐immunoreactive cells (neural stem/progenitor cells, NSPCs) were also detected, mainly surrounding the implanted cells (Figure 2a,c–h). However, on post‐MCAO day 14, although nestin‐immunoreactive cells were still present in the infarcted brain, the number of GFP‐immunoreactive cells decreased and cells became fragmented (Figure 2i–k). These findings confirmed the existence of transplanted EPO/EGFP/3T3 cells in the infarcted brain for at least 7 days; however, most of them did not survive for 14 days.

We also used ELISA to analyze the EPO concentration after EPO/EGFP/3T3 transplantation in bilateral striata on days 3, 7, and 14 post‐MCAO. In the infarcted striatum, the EPO concentration was higher on post‐MCAO day 3 compared with days 7 and 14 (1,284 ± 42 vs. 599 ± 169 and 185 ± 83 pg/mg; *p* < 0.05) (Figure 2b). In the contralateral striatum, the EPO concentration was not significantly different on post‐MCAO days 3, 7, and 14. These results imply that the implanted EPO/EGFP/3T3 cells expressed EPO for ~7 days after transplantation.

### 3.2 Increased intracerebral BDNF concentration after cell implantation

**Figure 3:**
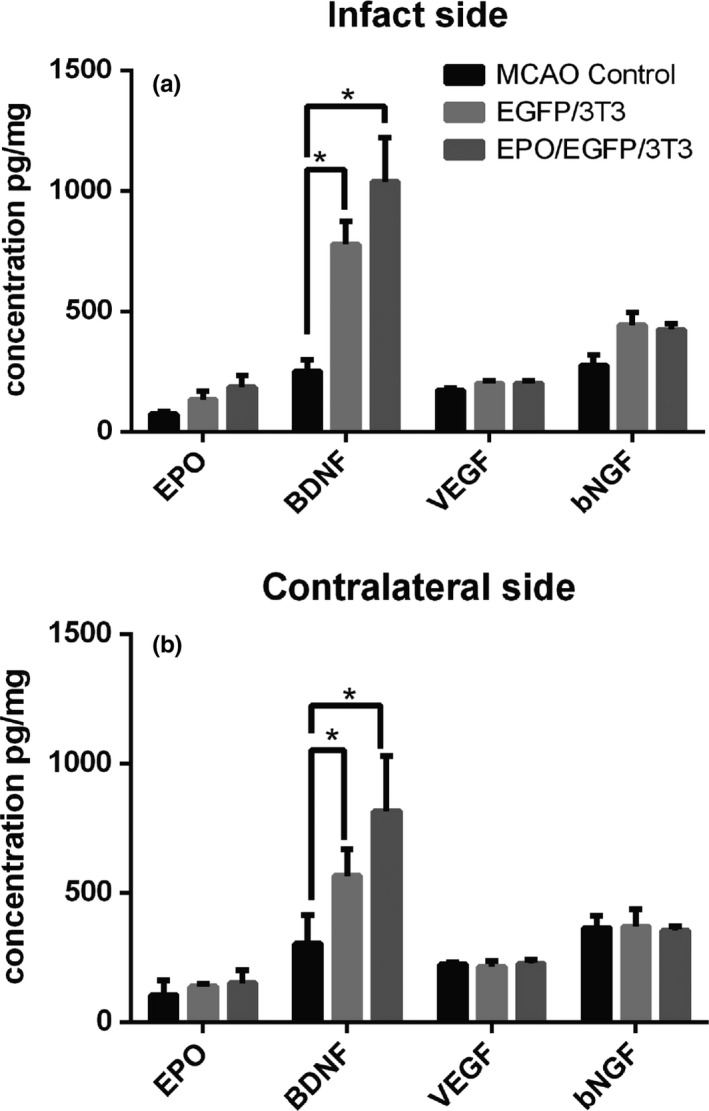
The concentration of erythropoietin (EPO), brain‐derived neurotrophic factor (BDNF), vascular endothelial growth factor (VEGF), and bNGF in bilateral striata on post‐middle cerebral artery occlusion (MCAO) day 14 was analyzed using enzyme‐linked immunosorbent assay (ELISA). The concentration of BDNF was significantly increased in (a) the infarcted and (b) the contralateral sides of EGFP/3T3‐ and EPO/EGFP/3T3‐treated rats compared with untreated MCAO control. The concentration of EPO, VEGF, and bNGF was similar among the groups (*n* = 3; *p < 0.05; **p < 0.01)

Previous studies have shown that 3T3 fibroblasts can secrete some growth factors, such as BDNF, VEGF, and NGF (Dudas et al., 2011; Saito et al., 2003; Young et al., 1975). Thus, we used ELISA to analyze the concentration of these trophic factors in bilateral striata 14 days after EGFP/3T3 or EPO/EGFP/3T3 transplantation. The concentration of NGF and VEGF was not different in the striatum of MCAO rats with or without EGFP/3T3 and EPO/EGFP/3T3 treatment (Figure 3). However, BDNF concentration was higher in the infarcted striatum of the EGFP/3T3 and EPO/EGFP/3T3 groups compared with the untreated group (778 and 1,038, respectively, vs. 250 pg/mg; *p* < 0.05). In addition, a higher BDNF concentration was detected in the contralateral striatum after treatment (565 and 815, respectively, vs. 303 pg/mg; *p* < 0.05). The BDNF concentration was similar between MCAO rats with EGFP/3T3 and EPO/EGFP/3T3 treatment in bilateral striata. The EPO concentration in bilateral striata was not different between the three groups on day 14 post‐MCAO. Taken together, these results showed that the post‐MCAO transplantation of fibroblasts (EGFP/3T3 or EPO/EGFP/3T3 cells) increased the concentration of BDNF in bilateral striata of MCAO rats.

### 3.3 EPO/EGFP/3T3 treatment enhanced cell proliferation and differentiation in the SVZ

**Figure 4:**
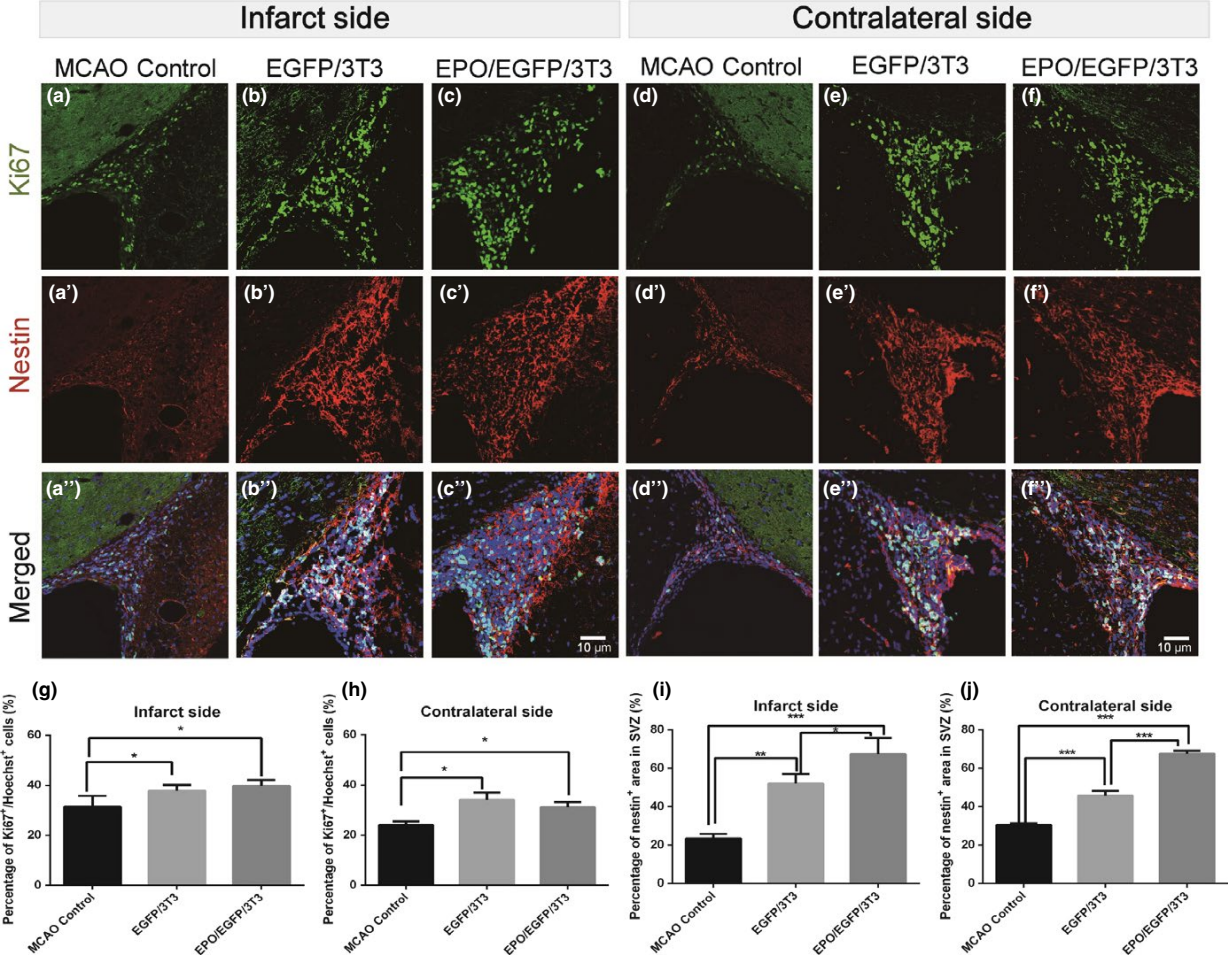
Proliferation of neural stem/progenitor cells in the subventricular zone (SVZ) after middle cerebral artery occlusion (MCAO). (a–f) Coronal sections of the forebrain of MCAO rats without treatment (a, d) or with EGFP/3T3 (b, e) or EPO/EGFP/3T3 (c, f) treatment were immunostained with an anti‐Ki‐67 antibody (green, proliferating cells) and an anti‐nestin (red, neural stem/progenitor cells) antibody, and counterstained with Hoechst 33342 (blue, nuclei). The density of Ki‐67‐immunoreactive cells was determined based on the number of Ki‐67‐positive cells/number of Hoechst‐positive cells within the SVZ on the infarcted side (a–c) and on the contralateral side (d–f). The density of nestin‐positive cells was determined based on the immunoreactive area of nestin‐positive cells/the area of the SVZ on the infarcted side (a–c) and on the contralateral side (d–f). The Ki‐67‐ or nestin‐immunoreactive area was higher in EPO/EGFP/3T3‐ and EGFP‐3T3‐treated rats than it was in untreated rats on the infarcted side (g, i) and on the contralateral side (h, j). Scale bar, 10 μm (*n* = 4 for each group; *p < 0.05; **p < 0.01; ***p < 0.001)

To investigate the cell proliferation capacity in the SVZ, we used immunohistochemistry to analyze the density of cells that were immunoreactive to Ki‐67 (a proliferating marker) and nestin (Figure 4). Intracerebral transplantation of either EPO/EGFP/3T3 or EGFP/3T3 cells increased the density of Ki‐67‐immunoreactive cells in the SVZ compared with the untreated MCAO control animals, not only on the infarcted side (39.8% ± 2.4% and 37.8% ± 2.2%, respectively, vs. 31.4% ± 4.3%; *p* < 0.05), but also on the contralateral side (31.1% ± 4.2% and 34.1% ± 5.7%, respectively, vs. 24.0% ± 3.1%; *p* < 0.05). The density of Ki‐67‐immunoreactive cells in the SVZ was similar between MCAO rats with EPO/EGFP/3T3 treatment and those with EGFP/3T3 treatment. EPO/EGFP/3T3 and EGFP/3T3 treatment also increased the area of nestin‐immunoreactive cells in the bilateral SVZ compared with the untreated control (infarcted side: 67.3% ± 8.4% and 51.9% ± 5.0%, respectively, vs. 23.4% ± 2.4%; *p* < 0.01; contralateral side: 67.5% ± 1.6% and 45.7% ± 2.4%, respectively, vs. 30.4% ± 0.9%; *p* < 0.01). Notably, the nestin‐immunoreactive area in bilateral SVZs was larger in EPO/EGFP/3T3‐treated versus EGFP/3T3‐treated MCAO rats.

**Figure 5:**
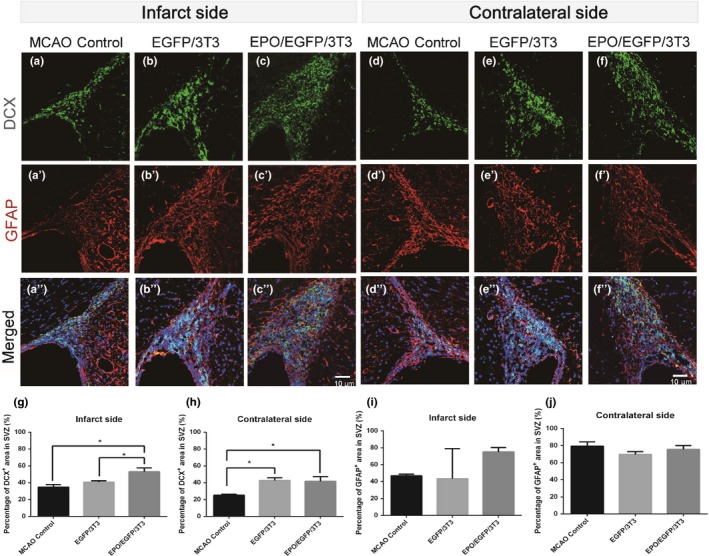
Differentiation of neural stem/progenitor cells in the subventricular zone (SVZ) after middle cerebral artery occlusion (MCAO). (a–f) Coronal sections of the forebrain of MCAO rats without treatment (a, d) or with EGFP/3T3 (b, e) or EPO/EGFP/3T3 (c, f) treatment were immunostained with an anti‐doublecortin (DCX) (green, neuroblasts) and an anti‐glial fibrillary acidic protein (GFAP) (red, astrocytes) antibody, and counterstained with Hoechst 33342 (blue, nuclei). The density of DCX‐ or GFAP‐positive cells was determined based on the immunoreactive area of DCX‐positive or GFAP‐positive cells/the area of the SVZ on the infarcted side (a–c) and on the contralateral side (d–f). The DCX‐immunoreactive area was larger in EPO/EGFP/3T3‐treated rats than it was in EGFP‐3T3‐treated or untreated rats on the infarcted side (g) and on the contralateral side (h). (i, j) The GFAP‐immunoreactive area was similar among the groups. Scale bar, 10 μm (*n* = 4 for each group; *p < 0.05)

We also analyzed the cells that were immunoreactive to DCX (a neuroblast marker) and GFAP (an astrocyte marker), to investigate the cell differentiation in bilateral SVZs (Figure 5). MCAO rats that received EPO/EGFP/3T3 treatment showed a larger DCX‐immunoreactive area in the SVZ than did EGFP/3T3‐treated and untreated MCAO rats on the infarcted side (53.1% ± 8.9% vs. 40.6% ± 3.1% and 34.6% ± 6.2%, respectively; *p* < 0.05). On the contralateral side, EPO/EGFP/3T3 and EGFP/3T3 treatment increased the DCX‐immunoreactive area compared with untreated MCAO rats (41.6% ± 11.2% and 42.5% ± 6.5%, respectively, vs. 25.0% ± 2.5%; *p* < 0.05). The GFAP‐immunoreactive area in bilateral SVZs was similar among the three groups. These findings indicate that both EPO/EGFP/3T3 and EGFP/3T3 treatments enhanced cell proliferation in the bilateral SVZs of MCAO rats. EPO/EGFP/3T3 treatment was also able to promote cell differentiation toward neuroblasts in bilateral SVZs.

### 3.4 EPO/EGFP/3T3 treatment reduced infarct size and improved functional recovery

**Figure 6:**
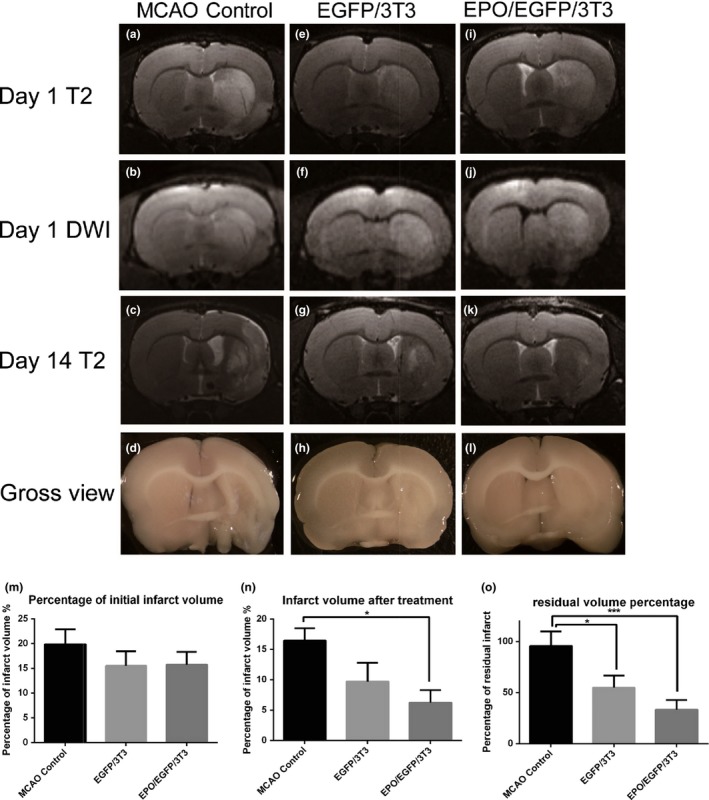
Assessment of infarct volume in middle cerebral artery occlusion (MCAO) rats using magnetic resonance imaging (MRI). Hyperintensity signals (cerebral infarct) were detected by T2‐weighted imaging and diffusion‐weighted imaging (DWI) on days 1 and 14 post‐MCAO in rats without treatment (a–c) or with EGFP/3T3 (e–g) or EPO/EGFP/3T3 (i–k) treatment. (m) The infarct volume is presented as the infarct size/whole brain size in each group. There was no significant difference in infarct volume between the groups on post‐MCAO day 1. (n) The residual infarct volume on post‐MCAO day 14 was smaller in EPO/EGFP/3T3‐treated rats than it was in untreated rats. (o) The ratio of the residual infarct volume over the initial infarct volume was lower in MCAO rats that received either EPO/EGFP/3T3 or EGFP/3T3 treatment compared with untreated MCAO rats (*n* = 3; *p < 0.05; ***p < 0.001)

To investigate the effect of EPO/EGFP/3T3 treatment on infarct volume, we used MRI to analyze the infarct size on days 1 and 14 post‐MCAO in rats with or without EPO/EGFP/3T3 and EGFP/3T3 treatment (Figure 6). On day 1 before treatment, the cerebral infarct was detected by both T2‐weighted imaging (Figure 6a–c) and DWI (Figure 6d–f). The infarct size before treatment was similar among the three groups, as measured using T2‐weighted imaging (Figure 6m). On day 14, however, the residual infarct volume in EPO/EGFP/3T3‐treated rats (Figure 6g–i) was significantly lower than that in untreated rats (6.2% ± 6% vs. 16.5% ± 6%; *p* = 0.02) (Figure 6n). We also analyzed the ratio of the residual infarct volume over the initial infarct volume and found that it was lower in MCAO rats that received either EPO/EGFP/3T3 or EGFP/3T3 treatment compared with untreated MCAO rats (33.4% ± 22.6% and 55.0% ± 28.4%, respectively, vs. 95.3% ± 35.6%; *p* = 0.0004 and 0.02) (Figure 6o).

**Figure 7:**
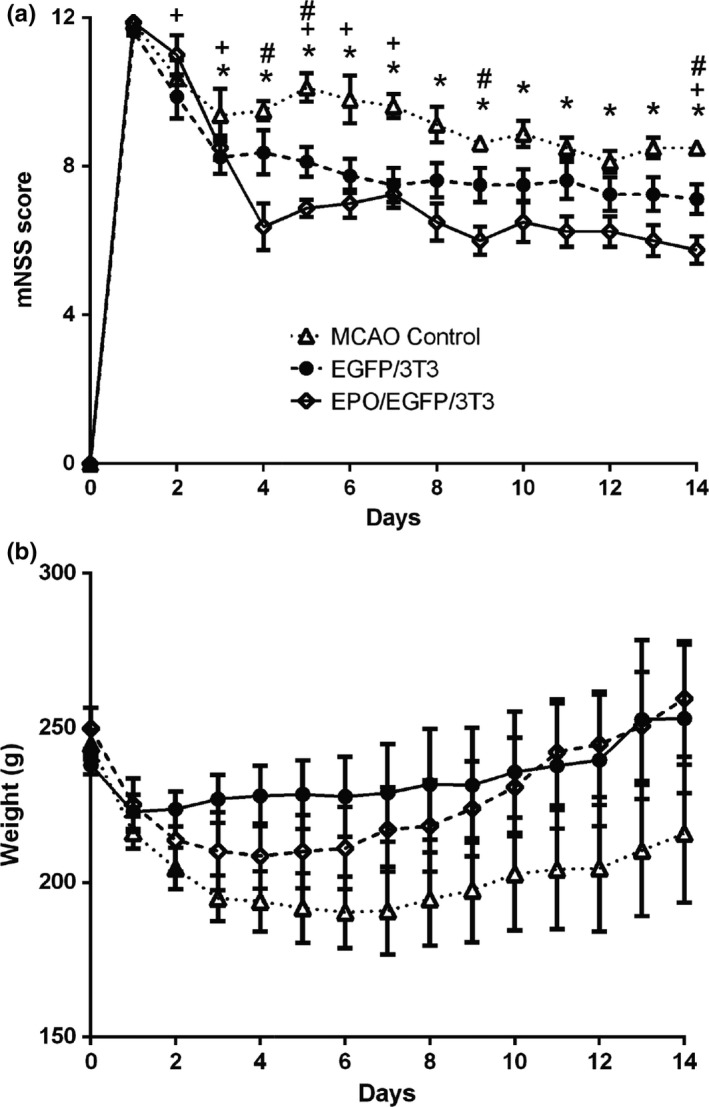
Behavior and body weight of middle cerebral artery occlusion (MCAO) rats. (a) The functional performance of MCAO rats was assessed using modified Neurological Severity Score (mNSS) evaluation from post‐MCAO days 0–14. MCAO rats that received EPO/EGFP/3T3 or EGFP/3T3 treatment had better functional recovery than did the untreated rats (*n* = 8 in each group; *p < 0.05 between the EPO/EGFP/3T3 and untreated control groups; +*p* < 0.05 between the EGFP/3T3 and control groups; #*p* < 0.05 between the EPO/EGFP/3T3 and EGFP/3T3 groups). (b) The body weight was similar among the groups

Finally, we evaluated the daily behaviors of MCAO rats with or without EPO/EGFP/3T3 and EPO/EGFP treatment within 14 days post‐MCAO (Figure 7). Based on mNSS functional assessment, EPO/EGFP/3T3‐treated MCAO rats exhibited lower mNSS scores (indicating a better functional performance) from day 3 to day 14 post‐MCAO compared with untreated MCAO rats (Figure 7a). EGFP/3T3 treatment improved post‐MCAO behaviors only at the early stage (<7 days). MCAO rats with EPO/EGFP/3T3 treatment also showed a better functional performance compared with MCAO rats that received EGFP/3T3 treatment on days 4, 5, 9, and 14 post‐MCAO. Conversely, body weight was similar among the three groups at 2 weeks post‐MCAO (Figure 7b).

## 4 DISCUSSION

Although EPO is a potential medication for acute ischemic stroke, its BBB penetration complicates the clinical usage of EPO via systemic delivery for stroke therapy. In this study, we directly transplanted EPO‐producing fibroblasts into the striatum of infarcted rat brains, to overcome this limitation of EPO. We demonstrated that intracerebral EPO/EGFP/3T3 treatment increased the expression of both EPO and BDNF in the infarcted brain. On post‐MCAO day 14, the number of Ki‐67‐, nestin‐, or DCX‐immunoreactive cells in bilateral SVZs was higher in EPO/EGFP/3T3‐treated versus untreated MCAO rats, indicating the enhancement of neurogenesis after EPO/EGFP/3T3 treatment. Most importantly, post‐MCAO EPO/EGFP/3T3 treatment significantly reduced infarct size and improved functional recovery. Therefore, the intracerebral transplantation of EPO‐producing fibroblasts benefited an ischemic stroke model, probably via the enhancement of neurogenesis.

EPO is a well‐known hematopoietic cytokine that has been widely used to treat patients with anemia (Subiros, Del Barco, & Coro‐Antich, 2012). Recently, EPO has been shown to enhance endogenous neurogenesis and improve functional recovery in a rodent model of ischemic stroke (Cho et al., 2010; Gonzalez et al., 2013; Nguyen et al., 2014; Siren et al., 2001; Wang et al., 2004). However, systemic delivery of EPO, which is the current clinical practice for anemia, did not consistently show effectiveness and safety in clinical trials of patients with stroke, probably because of the poor BBB penetration of EPO, which leads to the requirement of a high, but toxic, dose of EPO (Siren et al., 2001; Wang et al., 2004). In this study, after we singly transplanted EPO‐producing fibroblasts into the infarcted striatum, a high EPO concentration was detected in the infarcted brain for at least 1 week, implying the secretion of EPO from fibroblasts at the early stage of stroke. In addition, treatment with EPO‐producing fibroblasts was associated with a higher density of proliferating cells, NSPCs, or neuroblasts in the SVZ after cerebral infarct. These results were in accordance with previous reports that showed that poststroke EPO treatment enhanced not only the proliferation, but also the neuronal differentiation of NSPCs (Gonzalez et al., 2013). A previous study also showed that conditional EPO receptor knockdown reduced NSPC proliferation, migration, and neurogenesis in MCAO rats (Tsai et al., 2006). We also found numerous nestin‐immunoreactive cells appearing around the transplanted cells at the injection route. This result may imply that EPO also attracts NSPCs that migrate toward the infarcted region at the injection site.

Genetically modified cells, such as immortalized cells, can offer a local sustainable intracerebral delivery of trophic substances, to rescue injured neurons (Jin, Fischer, Tessler, & Houle, 2002; Rossner et al., 1996). Previous studies have demonstrated that NIH‐3T3 fibroblasts were less sensitive to contact inhibition and are less likely to induce tumorigenesis (Jainchill, Aaronson, & Todaro, 1969). These transplanted 3T3 fibroblasts were also able to survive for 2–8 weeks in the brain or spinal cord of rodent models of intracerebral hemorrhage, Parkinson's disease, and spinal cord injury, with various beneficial effects (Grandoso et al., 2007; Jeon, An, Kim, Park, & Lee, 2008; Jin et al., 2002; Liu, Himes, Murray, Tessler, & Fischer, 2002; Rossner et al., 1996). Thus, the 3T3 fibroblasts were carrier cells that were appropriate for intracerebral transplantation. However, we found that most of our implanted fibroblasts survived less than 14 days, and that the concentration of EPO was reduced to the baseline level on post‐MCAO day 14, which may result from the harsh environment of the infarcted brain, in which the transplanted cells were surrounded by inflammatory cells and hypoxic conditions (Savitz, Dinsmore, Wechsler, Rosenbaum, & Caplan, 2004).

Fibroblasts secrete several neurotrophic factors, such as BDNF, VEGF, and NGF (Dudas et al., 2011; Saito et al., 2003; Young et al., 1975). In both EPO/EGFP/3T3‐ and EGFP/3T3‐treated MCAO rats, we found that the concentration of BDNF, but not of VEGF or NGF, was elevated in the infarcted brain. BDNF is a neurotrophic factor that plays an important role in endogenous neurogenesis in physiological conditions and in the presence of neurological diseases (Wang et al., 2004). Although the post‐MCAO functional improvement was better and the neuronal differentiation capacity was higher in EPO/EGFP/3T3‐ versus EGFP/3T3‐treated rats, EGFP/3T3 treatment still enhanced the proliferation of NSPCs and partially improved the behavior performance of MCAO rats. Therefore, the transplantation of fibroblasts alone (without EPO effects) may benefit MCAO rats regarding functional recovery, possibly through the promotion of NSPC proliferation via BDNF expression. Previous studies also showed that fibroblast treatment can stimulate the proliferation of cultured vascular endothelial cells and enhance neuroblast migration in a rodent model of intracerebral hemorrhage (Chen et al., 2012). The EPO‐producing fibroblasts can thus secret BDNF and EPO simultaneously in the infarcted area, which enhanced the post‐stroke neurogenesis in a highly effective way. Therefore, the use of fibroblasts as carriers for cell therapy may produce a target protein via the transgenic approach and present the advantage of trophic support factors secreted by the fibroblasts.

The EPO/EGFP/3T3‐treated MCAO rats showed higher proliferating and neuronal differentiation capacities of NSPCs in the SVZ, which were accompanied by a better functional recovery and smaller infarct size compared with untreated MCAO rats. Endogenous neurogenesis contributes to poststroke functional recovery, as conditional ablation of mouse NSPCs reduced poststroke motor and cognitive improvement (Jin, Wang, Xie, Mao, & Greenberg, 2010). In patients with subarachnoid hemorrhage (a subtype of stroke), we recently demonstrated that the increase in NSPC proliferation‐promoting factors in the cerebrospinal fluid (CSF) was associated with better functional outcomes (Y. A. Chen, Wang, Liu, Young, & Tsai, 2018). Taken together, these results suggest that the post‐MCAO behavioral improvement observed in EPO/EGFP/3T3‐treated rats results from the enhancement of NSPC proliferation and neuronal differentiation.

After single unilateral fibroblast implantation at the striatum on the infarcted side, intracerebral BDNF concentration was increased and NSPC proliferation was facilitated in the contralateral hemisphere. As there is no brain–CSF barrier and proteins are able to diffuse throughout the brain via the CSF (Chen et al., 2012), we postulated that the BDNF produced in the infarcted brain might diffuse toward the contralateral hemisphere and stimulate the proliferation of NSPCs at the contralateral SVZ.

Although transplantation of modified autologous fibroblasts may reduce the possibility of immune reaction following intracerebral injection, the time requiring cell harvest, cell expansion, and genetic modification is too long, impeding cell transplantation at the acute stage of stroke. Therefore, allogenic transplantation may be more appropriate for future application. While the transplanted allogenic fibroblasts were eliminated within 14 days after MCAO in our experiment, the transplanted cells have already released sufficient trophic factors for enhancement of neurogenesis and functional recovery in MCAO rats. As the result, transplantation of genetic modified allogenic fibroblasts may be tested at first in the future.

## 5 CONCLUSION

We demonstrated that intracerebral treatment with EPO‐producing fibroblasts (as carriers) can reduce infarct size and improve poststroke functional recovery. These benefits may stem from the enhancement of NSPC proliferation and neuronal differentiation via the effect of EPO and BDNF secreted from fibroblasts. This study contributes to future research on EPO‐related therapy in stroke and fibroblast‐assisted cell therapy in neurological diseases.
