# Supplementary material for: Intracerebral transplantation of erythropoietin‐producing fibroblasts facilitates neurogenesis and functional recovery in an ischemic stroke model
Source: Brain Behav. 2019 Mar 28;9(5):e01274. doi: 10.1002/brb3.1274 (PMC6520520; doi:10.1002/brb3.1274)
Supplement: Supplementary file 1 [file BRB3-9-e01274-s001.docx]

**Supplementary data**

Neurological function was graded on a scale of 0 to 18, which composite motor, sensory reflex and balance tests. It is based on the previous study. (J. Chen et al., 2001).

| **Modified Neurological Severity Score Point** | | | |
| --- | --- | --- | --- |
| Motor tests | | |  |
|  | Raising rat by tail | | 3 |
|  |  | Flexion of forelimb | 1 |
|  |  | Flexion of hindlimb | 1 |
|  |  | Head moved >10 to vertical axis within 30 s | 1 |
|  | Placing rat of floor (normal=0; maximum=3) | | 3 |
|  |  | Normal walk | 0 |
|  |  | Inability to walk straight | 1 |
|  |  | Circling toward paretic side | 2 |
|  |  | Falls down to paretic side | 3 |
| Sensory tests | | | 2 |
|  | Placing test (visual and tactile test) | | 1 |
|  | Proprioceptive test (deep sensation, pushing paw against table edge to stimulate limb muscles) | | 1 |
| Beam balance tests (normal=0; maximum=6) | | | 6 |
|  | Balances with steady posture | | 0 |
|  | Grasps side of beam | | 1 |
|  | Hugs beam and 1 limb falls down from beam | | 2 |
|  | Hugs beam and 2 limbs fall down from beam, or spins on beam (>60 s) | | 3 |
|  | Attempts to balance on beam but falls off (>40 s) | | 4 |
|  | Attempts to balance on beam but falls off (>20 s) | | 5 |
|  | Falls off; no attempt to balance or hang on to beam (20 s) | | 6 |
| Reflex absence and abnormal movements | | | 4 |
|  | Pinna reflex (head shake when auditory meatus is touched) | | 1 |
|  | Corneal reflex (eye blink when cornea is lightly touched with cotton) | | 1 |
|  | Startle reflex (motor response to a brief noise from snapping a clipboard paper) | | 1 |
|  | Seizures, myoclonus, myodystony | | 1 |
| Maximum points | | | 18 |
| One point is awarded for inability to perform the tasks or for lack of a tested reflex: 13-18, severe injury; 7-12, moderate injury; 1-6, mild injury. | | | |
